# Acceptability of a head-mounted assistive mouse controller for people with upper limb disability: An empirical study using the technology acceptance model

**DOI:** 10.1371/journal.pone.0293608

**Published:** 2023-10-31

**Authors:** Mohammad Ridwan Kabir, Hasan Mahmud, Md. Kamrul Hasan

**Affiliations:** 1 Systems and Software Lab (SSL), Islamic University of Technology (IUT), Boardbazar, Gazipur, Bangladesh.; 2 Department of Computer Science and Engineering, Islamic University of Technology (IUT), Boardbazar, Gazipur, Bangladesh.; Universidad Central de Chile, CHILE

## Abstract

Due to limited motor capabilities, people with upper limb disabilities have trouble utilizing a typical mouse while operating a computer. Different wearable Assistive Mouse Controllers (AMCs) have been developed to overcome their challenges. However, these people may not be able to realize the importance, ease of use, and social approval of these AMCs due to their fear of new technology, lack of confidence, and lack of ingenuity. These may negatively affect their attitude and intention toward accepting AMCs for equitable human-computer interaction. This study presents the development of a sensor-based head-mounted AMC, followed by an empirical analysis of its acceptance using the Technology Acceptance Model (TAM) from the socioeconomic perspective of Bangladesh. In a similar vein, we examined the effects of three additional psychological constructs—technology anxiety, confidence, and innovation, on its acceptance along with the original components of the TAM. A total of 150 individuals with stroke-induced upper limb disability participated in an online survey, and their responses were analyzed using confirmatory factor analysis and structural equation modeling, following the general least square method. Analysis revealed, about 96.44% of the participants had positive attitude towards the AMC, and almost 88.56% of them had positive intentions to accept it. Furthermore, about 68.61% of them expressed signs of anxiety, 96.35% were confident, and 94.16% of them had an innovative mindset in terms of device usage. The findings imply that individuals with an innovative mentality are more capable of comprehending the practical implications of a new technology than those without one. It is also feasible to reduce technological anxiety and boost a user’s confidence while using an AMC by combining an innovative mentality with straightforward device interaction techniques. Additionally, peer encouragement and motivation can significantly enhance their positive attitude towards accepting the AMC for facilitating their interaction with a computer.

## Introduction

Recent technological advancements have increased our dependency on computers for any interaction as simple as “*checking today’s date*” to a task as complex as “*writing a computer program*” [1]. Healthy people, due to their physical capability, can move the cursor on the computer screen and actuate mouse clicks with the help of a generic computer mouse. However, the scenario is quite different for people with disabilities of the upper limb, caused due to neurological disorders [2–5], accidents [6], or birth defects [7]. Compared to healthy individuals, people with upper limb disability can perform only a limited number of motor activities with their upper limbs, otherwise known as residual capabilities [8]. As a result, their participation in different motor activities is restricted, making them dependent on special devices, otherwise known as Assistive Technologies (ATs) [9]. Since a generic computer mouse is hand-held, such people require different kinds of ATs to facilitate alternative input modalities for interacting with a computer [9]. Hence, any AT that functions as an alternative to a generic computer mouse or a similar pointing device, allowing a disabled individual to interact with a computer, may be termed an Assistive Mouse Controller (AMC).

Stroke is the major cause behind upper limb disability in the socioeconomic context of Bangladesh [10–12]. Unfortunately, there is no comprehensive system to recognize the requirements of AMCs for the disabled community, let alone register their usage [13–16]. Moreover, since the majority of these people live below the poverty line [17], they are unable to afford [18] many of the existing state-of-the-art AMCs [19–35]. Consequently, they are not only falling behind in economically empowering themselves through activities involving a computer but also in contributing to the national economy of Bangladesh [36]. This suggests that there is a scope for developing an AMC for economically empowering the upper limb disabled community of Bangladesh. Considering their lack of experience in using AMC technology [16, 18], it is also imperative to study the probable influence of their perception of its *usefulness*, *ease of use*, *social approval*, etc., on its acceptance [37] from the socioeconomic perspective of Bangladesh. In a similar vein, it is very natural for them to exhibit signs of *anxiety* about the *ergonomics*, *complexity of interaction techniques*, *hygiene issues*, etc., associated with the technology [37–39]. Moreover, they might also feel less—*confident* [37, 40–42], *innovative*, *enthusiastic*, etc. [37, 43–45], while using the AMC technology. Moreover, analysis of these psychological constructs may provide insights into whether to *add new* or *modify existing* features of an AMC to favor its acceptance in the socioeconomic context of Bangladesh. However, a theoretical understanding of such influence on newly developed AMC technologies is inadequate in the literature [37, 46].

To date, researchers have explored the feasibility of various wearable sensors in the design and development of AMCs for people with upper limb disability while leveraging their residual motor capabilities [8, 19–34]. Among these capabilities, head movement is a natural, effective, and one of the most common modalities for moving a cursor [19–22]. Other alternatives include but are not limited to—tongue muscles movement [23], eye gaze tracking using eye-trackers, webcams, or other imaging sensors [24–26], Electromyography (EMG) [27, 28], Electrooculogram (EOG) [29–34], Brain-Computer Interfaces (BCI) [35], etc. However, to the best of our knowledge, an AMC for the upper limb disabled community from the socioeconomic perspective of Bangladesh has not yet been developed [13–16].

The Technology Acceptance Model (TAM) [40, 41] has been widely used by researchers [37–39, 43, 44, 46–58] for analyzing the influence of various psychological constructs, for example—Perceived Usefulness (PU), Perceived Ease of Use (PEU), Subjective Norm (SN), Attitude Towards Usage (ATU), Behavioral Intention (BI), etc., on the user’s perception and intention to use various ATs in general. It is also evident from the literature that the constructs external to the TAM, namely—Technology Anxiety (TA) (*perceived fear of a new technology*) [38, 39], Perceived Behavioral Control (PBC) (*perceived confidence of carrying out a task with a technology*) [40–42], and Personal Innovativeness (PI) (*portraying enthusiasm, curiosity, innovation, etc., while using a new technology*) [43–45], also play a crucial role behind the acceptability of different ATs [37, 47, 48, 59]. However, to the best of our knowledge, the influence of these external constructs along with the internal ones of the TAM on the acceptance of AMCs by individuals with upper limb disability from the socioeconomic perspective of Bangladesh has not been carried out.

From the perspectives mentioned above, in this study, we present the design and development of a functional prototype of a head-mounted sensor-based AMC to facilitate equitable human-computer interaction for individuals with stroke-induced upper limb disability. With motivation from prior studies [19, 20, 22] the proposed AMC utilizes an Inertial Measurement Unit (IMU) to register head motion for moving a mouse cursor on the screen. For registering mouse clicks, on the other hand, we adapted the idea of exploiting cheek muscle twitches from [8]. However, the authors in [8] leveraged flex sensors for registering the cheek muscle twitches, whereas, in our study, we used infrared sensors. The rationale behind doing so stems from the fact that the flex sensors had to be attached to a user’s cheek with the help of adhesives [8]. We perceived that this may significantly reduce the ease of use, comfort, and wearability of the device. Although we were able to overcome these limitations by using infrared sensors, we were faced with new challenges related to fluctuating sensor readings due to—ambient light, varying shapes of cheek muscles, etc. Special design considerations from both hardware and software perspectives were taken into account to address these challenges. Thus, it is safe to say that such an AMC for the upper limb disabled community from the socioeconomic perspective of Bangladesh is the first of its kind. From a similar standpoint, we also examine the influence of the fundamental psychological constructs of the original TAM framework (PU, PEU, SN, ATU, and BI) [39, 40] as well as the three constructs external to it, namely—TA [38, 39], PBC [40–42, 54, 55, 60] and PI [43–45, 56, 61–63], on the users’ perceptions of and intentions to use the proposed AMC for the previously mentioned reasons.

To summarize our contributions to this study—(1) to develop an IMU and infrared sensor-based wearable AMC for people with upper limb disability from the socioeconomic perspective of Bangladesh, and (2) to analyze the impact of different psychological constructs of the TAM framework (PU, PEU, SN, ATU, and BI) [40, 41] along with three external constructs (TA, PBC, and PI) adapted from prior studies [38–45, 49–56, 60–63] on one another (significant or otherwise) on the acceptability of the proposed AMC to people with upper limb disability in a similar vein.

## Theoretical background

**Fig 1:**
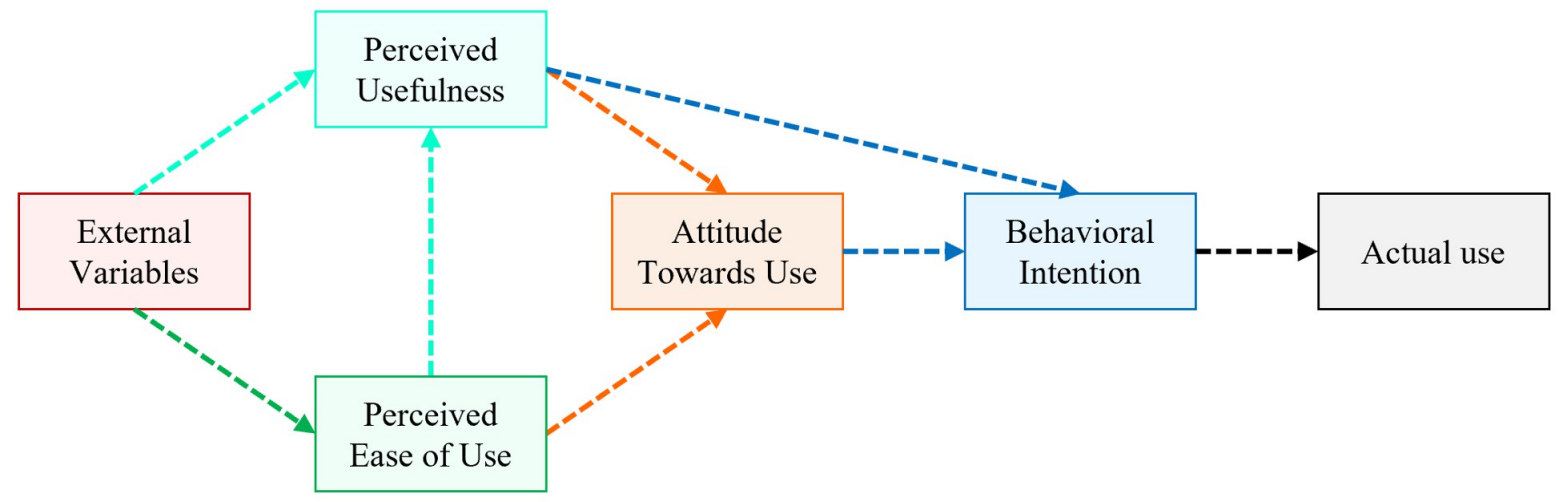
Outline of the Technology Acceptance Model (TAM).

The Technology Acceptance Model (TAM) [40–42], depicted in Fig 1, has been widely used for analyzing users’ acceptance of different ATs in previous studies [37–39, 43, 44, 47–56]. According to the literature [40–42], the influences of different psychological constructs of the TAM on the users’ acceptance of new technology are defined as follows -

- **Perceived Usefulness (PU)**: “*The degree to which a person believes that using a particular system would enhance his or her job performance*” [40–42].
- **Perceived Ease of Use (PEU)**: “*The degree to which a person believes that using a particular system would be free of effort*” [40–42].
- **Subjective Norm (SN)**: “*An individual’s perception that most people who are important to him or her think s/he should or should not perform the behavior in question*” [40–42].
- **Attitude Towards Usage (ATU)**: “*An individual’s positive or negative feelings (evaluative affect) about performing the target behavior*” [40–42].
- **Behavioral Intention (BI)**: “*A measure of the strength of one’s intention to perform a specified behavior*” [40–42].

The analysis of users’ acceptance of technology using the TAM framework typically involves a self-administered, closed-ended, *n*-point (*n* = 4, 5, 6 or 7) Likert scale-based survey questionnaire, which is divided into sections that represent different psychological constructs (e.g., PU, PEU, SN, etc.) [39, 42, 55, 64, 65]. Each question corresponding to a particular construct is normally referred to as a measurement *item*. Based on a theoretical analysis of the possible influence of one construct over another, alternative hypotheses are postulated [42, 49, 66, 67] and later tested (*rejected* or *accepted*) during data analysis. In studies related to technology acceptance, involving TAM [38, 39, 49, 50, 52, 66, 67], data analysis typically involves *two* steps—1) developing a Measurement Model and 2) developing a structural model using Structural Equation Modeling (SEM) [39, 61, 66]. A brief description of these two steps is provided in the subsequent paragraphs.

### Measurement model

The measurement model is generally developed through Exploratory Factor Analysis (EFA) or Confirmatory Factor Analysis (CFA) [39, 66, 68–70]. Detailed descriptions of EFA and CFA are beyond the scope of this study, and the reader is requested to refer to the work of Noora Shrestha [70] for better comprehension. However, before either EFA or CFA could be conducted for developing the measurement model, it is necessary to check whether the dataset under consideration is adequate for factor analysis. The adequacy test involves both Bartlett’s test of sphericity and Kaiser-Meyer-Olkin (KMO) measure of sampling adequacy [69, 70]. Bartlett’s test of sphericity is intended to test whether the items in the questionnaire are correlated enough, such that the correlation matrix does not become an identity matrix. This can be verified if the value of *p*, obtained from this test is less than 0.05 at 95% confidence interval [70]. The KMO measure, on the other hand, is used to determine whether the sample size is large enough for factor analysis. The KMO value ranges between 0 and 1, indicating 4 types of sample size, namely—“*adequate*” (0.80≤ KMO <1.00), “*middling*” (0.70≤ KMO <0.79), “*mediocre*” (0.60≤ KMO <0.69), and “*inadequate*” (KMO <0.60) [70]. Factor analysis cannot be carried out with *inadequate* sample size, in which case the sample size must be increased [70]. The measurement model is developed to test the following properties -

- **Overall reliability of the questionnaire**: Measured using the Cronbach’s Alpha (*α*) (CA) test, where *α* > 0.7 is recommended for acceptable reliability [39, 66].
- **Internal consistency of the constructs**: Generally measured using their respective Composite Reliability (CR) [39, 61, 66, 68, 71], where the typical value of CR should be greater than 0.7 [39, 68]. However, CR>0.6 and CR≤0.7 is also acceptable [66, 71].
- **Individual item reliability**: Measured using factor loadings (λ), where factors are defined as latent or unobserved variables that affect a particular construct [39, 61, 68, 71, 72]. Only the factors with eigenvalues greater than 1 are considered for analysis [70]. Although the typical value of λ should be greater than 0.7, values between 0.5 and 0.7 are also acceptable [68, 72].
- **Convergent Validity (CV) of a construct**: An indicator of high correlation between theoretically related items. Alternatively, CV ensures that the items intended for measuring a construct are indeed measuring that construct. For ensuring the CV of a construct, both the CR and the Average Variance Extracted (AVE) of that construct are considered. Previous studies suggest that CR>0.7 and AVE>0.5 combined, are indicators of good convergent validity [39, 70, 71, 73]. However, if AVE<0.5, but CR>0.6, the CV of the construct is still considered to be adequate [71].
- **Discriminant Validity (DV) of a construct**: Ensures that the items of a particular construct are not measuring a different construct [39, 68]. DV can be tested using the Fornell and Larcker criterion [45], where the squared root of AVE for a construct (usually placed on the diagonal of the construct correlation matrix) is greater than its correlation coefficients with other constructs [38, 45, 61, 66, 68, 71].

### Structural model

After a measurement model has been developed, Structural Equation Modeling (SEM) is used to test the hypotheses postulated earlier during the theoretical analysis of the possible influence of one construct over another, relevant to the acceptance of technology [38, 39, 49, 50, 52, 66–68], for developing a structural model. For example, if we consider *two* constructs, *CONS*_1_ and *CONS*_2_, where *CONS*_1_ is hypothesized to have an influence on *CONS*_2_; the influence is represented with an arrow, termed as a *path*, from *CONS*_1_ to *CONS*_2_. The standardized *β* coefficient, otherwise known as the *path coefficient*, is considered as the magnitude of the influence. This coefficient may be estimated using any one of the following methods—Partial Least Square (PLS) [39, 66], Maximum Likelihood [52, 74, 75], Unweighted Least Squares (ULS) [76–78], and Generalized Least Squares (GLS) [77]. The statistical significance of the path coefficient (*β*) is determined either through bootstrapping [61, 74, 79], *t*-tests, or Z-tests [66] to calculate *p*-values at a significance level of 0.05. Therefore, a hypothesis is *accepted*, if the *z*-value is either <−1.96 or >1.96 and *p* < 0.05, otherwise, it was *rejected*. After quantitatively investigating all the hypotheses, the results are combined to generate a path model as an outcome of SEM. Although the path model gives an overview of the influences (*significant* or *insignificant*) of one construct over another, it is important to investigate the relative fit of the data to the model. For this purpose, many studies have recommended fit indices, namely—the ratio of chi-square to degrees of freedom (*χ*^2^/df), the Goodness-of-Fit Index (GFI), Adjusted Goodness-of-Fit Index (AGFI), Comparative Fit Index (CFI), Tucker-Lewis Index (TLI), Normed Fit Index (NFI), and Root-Mean-Square Error of Approximation (RMSEA) [49, 67, 69, 80, 81].

## Literature review

Till date, several researchers have leveraged TAM for analyzing how different psychological constructs, under the influence of external variables, affect user’s acceptance of different technologies. Liu et al. [46] tested 7 commercially available wearable ATs for motor function rehabilitation, daily activity assistance, and knee care for the elderly (aged 65 and above). They analyzed 14 perception variables related to device usage [PU, PEU, SN, ATU, TA, ease of maintenance (EOM), weight (WEI), portability (POR), perceived comfort (PCOM), perceived convenience (PCON), appearance (APP), perceived need (PN), perceived cost-effectiveness (PCE), and trust (TRU)] by testing 27 hypotheses. They recruited healthy and educated older adults who did not have much need for ATs in general for their survey. After gathering demographic data via a questionnaire, the participants were informed about the functions, features, and history of the devices. They could use any three of the seven gadgets to complete three tasks with each one, followed by rating their perception of using the gadgets on a 7-point Likert scale. The findings of their study suggest that developers should understand the need of the stakeholders while ensuring simple interaction mechanisms and intuitive and self-explanatory UI. This may significantly increase PEU, device effectiveness, and user satisfaction while reducing the probability of errors. Most importantly, reliable quality and functional stability must be ensured to positively affect the constructs—TRU and PU. Finally, the user base of such devices should be increased to reduce the feeling of shame or shyness about using them. Apart from making proper arrangements for advertising the existence, usage, and benefits of using these devices, mental health counseling sessions can also be arranged for achieving these goals. Liu et al. in another study [57], explored whether and why patients accept a Health Information Technology (HIT) platform and continue to accept it over time. They conducted the study on patients with type-2 diabetes and hypertension using a patient-focused, touchscreen tablet-based system over a 24-week implementation period. They were motivated by the fact that previous studies only considered conventional constructs while analyzing acceptance of HIT platforms, whereas many nonconventional and inferred constructs might influence their acceptance as well. Moreover, the prior studies mostly explored smartphone-based applications and telemedicine, and hence, the findings of those studies might not apply to HIT systems designed for the self-management of chronic diseases at home. The authors proposed a theoretical model for HIT acceptance by patients with chronic diseases performing self-management at home while additionally considering the psychological constructs—perceived hand function (PHF), perceived visual function (PVF), and perceived space adequacy (PSA), external to the TAM framework. Tsai et al. [39], in addition to the traditional components of TAM, conducted a study with 31 older patients with cardiovascular diseases and 81 older adults in general, to understand the behavioral effects of TA, Perceived Ubiquity (PUB), and Resistance to Change (RC), on the adoption of a wearable cardiac warming system in older adults. Their research findings state that TA has negative effects on PEU and PUB, while PUB affects both PU and PEU of the cardiac warming system. On top of these, PU was found to have an indirect effect on BI through ATU. Felea et al. [66] analyzed the influence of constructs—Perceived Enjoyment (PE), defined as, “*the level to which using a specific technology or service is seen as enjoyable*”, and Visual Attractiveness (VA), defined as, “*an aesthetic product design expressed through shapes, colors, and materials and user interfaces such as device menus and the mobile applications of wearable devices*”, on the adoption of wearable technologies among 192 Romanian students using the TAM. Their analysis revealed that apart from the relation between the original constructs of the TAM, VA positively affects PE and ATU, while PE positively affects PU, ATU, and BI when it comes to the adoption of wearable technologies. Ashfaq et al. [49] analyzed external constructs, namely—Perceived Irreplaceability, Perceived Credibility, Compatibility, etc., that might influence elderly diabetic peoples’ intention to continue using digital health wearables through a survey from 223 diabetic patients, aged 60 years and above. The findings of their study revealed that all the constructs mentioned above had a positive influence on the intention to continue using digital health wearables. Lin et al. [38], have developed an instrumented wearable vest for monitoring the quality of posture among elderly people. They identified Technology Anxiety (TA) as a common psychological trait among elderly people when acceptance of new technology is of concern. About 50 elderly people were recruited for their study and leveraging TAM, they analyzed the ATU and BI of their proposed technology under the influence of the psychological constructs—TA, PU, and PEU. Hong et al. [51], Chuah et al. [52] and Kim et al. [67], through a survey involving 276, 226, and 363 participants, respectively, utilized TAM to empirically identify Visibility (VIS), Affective Quality (AQ), Relative Advantage (RA), Mobility (MB), Availability (AV), Subcultural Appeal (SA), Consumer Innovativeness (CI), etc., as potential external constructs that might influence adoption of smartwatches. The results of these studies suggest that the variables AQ and RA influenced PU, while MB and AV influenced PEU, and the variables, CI, SA, and VIS, were found to be significant indicators of ATU and BI of smartwatch adoption. Lunney et al. [50] deployed the TAM for gaining insights into a user’s perception of Wearable Fitness Technologies (WFT) and to analyze the relation between perceived health benefits and the use of WFTs. From their analysis, it may be stated that WFTs that have enhanced PU and PEU, are more likely to instigate increased positive ATU and BI towards their adoption. Apart from these technologies, evidence of studies on the acceptance of industrial upper limb exoskeleton [82, 83] and head-mounted display-based augmented reality [84] can also be found in the literature. A generic finding of these studies suggests that to increase the usage and adoption of ATs, they should be designed from both engineering and user perspectives while ensuring ease of use, comfort, usefulness, reliability, ergonomics, etc.

## Prototype development

**Fig 2:**
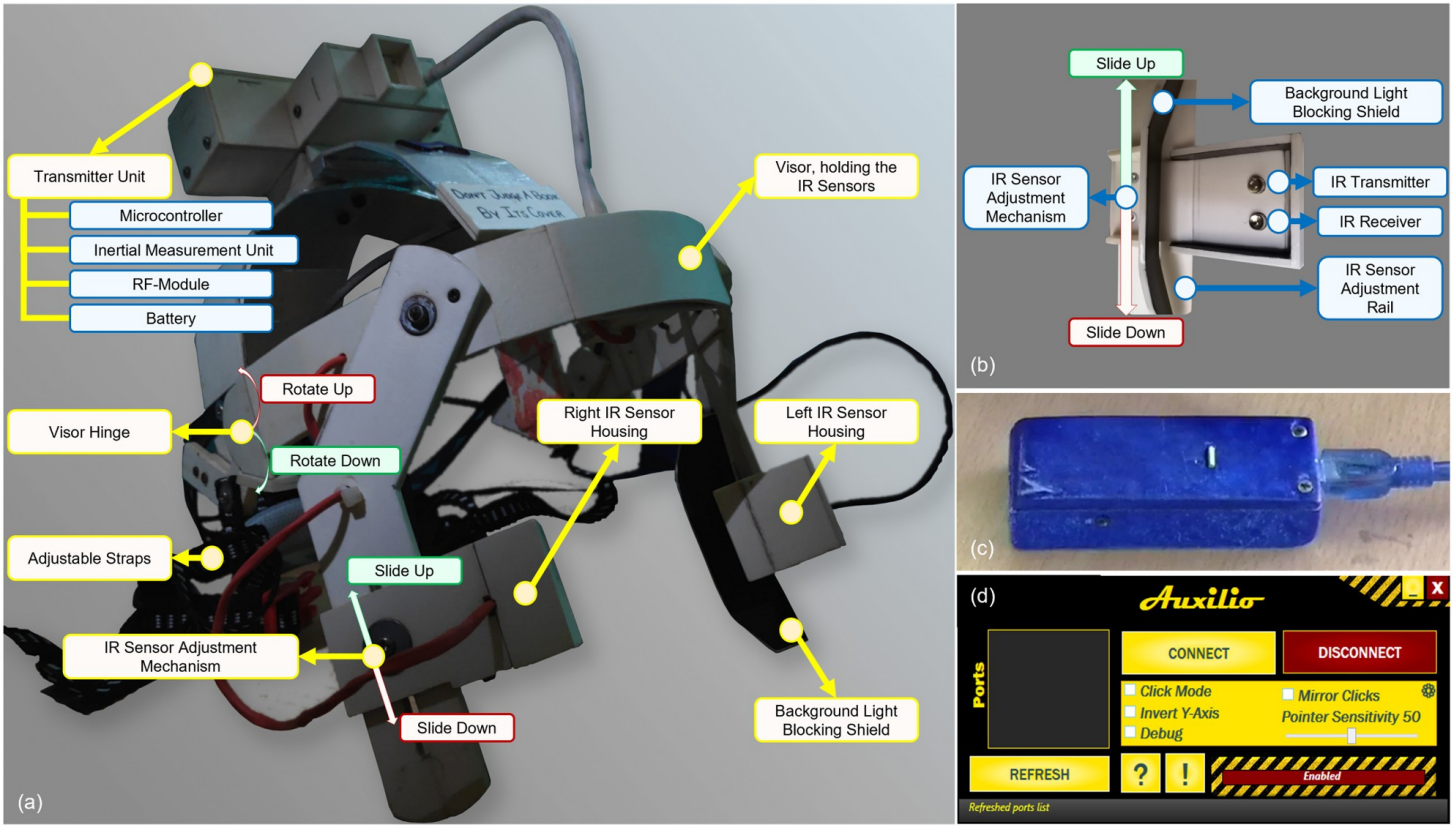
The constituent elements of the proposed Assistive Mouse Controller (AMC). (a) The wearable transmitter unit, for sensor data acquisition and transmission, (b) infrared sensor housing and adjustable mechanism, (c) the receiver unit, for wireless retrieval of sensor data and forwarding those to the device driver software, and (d) the custom device driver software for mapping sensor data to system calls for mouse control.

We have developed a working prototype of the proposed head-mounted AMC, as shown in Fig 2. It mainly consists of three separate entities—1) a transmitter unit, 2) a receiver unit, and 3) a device driver software.

The physical form factor of the *transmitter unit* is similar to that of a helmet, hence the AMC is head-mounted. It contains all the sensors, microcontroller, wireless communication module, and power source and is responsible for sensor data acquisition, processing, and wireless data transmission to a PC via the *receiver unit*. It is important to note that the form factor of the human head is not absolute, rather it varies across humans. Therefore, to ensure the comfort and wearability of the AMC by people with varying head sizes, adjustable head-straps have also been facilitated.

To facilitate mouse-cursor movement, we exploited absolute movements of the head. An Inertial Measurement Unit (IMU), featuring an MPU9250 (a combination of a 3-axis accelerometer, a 3-axis gyroscope, and a 3-axis magnetometer) was used to measure the *yaw* (horizontal rotation of the head about the z-axis) and the *pitch* (vertical rotation of the head about the y-axis) angles of head rotation in degrees. These values were then converted to 2*D* screen coordinates facilitating horizontal and vertical movements of the mouse cursor on the screen, respectively. A user can simply rotate his/her head by only ±15° both horizontally and vertically, and move the cursor about the entire screen. Literature suggests that head movements to this extent are well within the ergonomic range of motion of the human head and are unlikely to cause neck pains, in the long run, [85].

The transmitter unit contains a visor-like mechanism that can be rotated up and down to make it easier for the user to wear the AMC, as shown in Fig 2a. To facilitate left and right mouse-click actuation, we leveraged left and right cheek muscle twitches, respectively. One pair of infrared sensors (transmitter and receiver) per cheek (left and right), as depicted in Fig 2b, were used in this regard. Although the infrared sensor readings are inherently subject to fluctuations due to changes in the ambient lighting conditions, we have compensated for this limitation with the help of a strategically placed mechanical ambient light-blocking shield on the wearable itself as well as through software. Another inherent challenge with actuating mouse clicks in this manner is the variations in cheek shape from person to person, resulting in different patterns of cheek muscle movements. Therefore, to ensure proper actuation of mouse clicks, the infrared sensors are placed on the visor mechanism with vertically adjustable housing which can be slid up and down, as depicted in Fig 2b, allowing 2 degrees of adjustments to fit differently shaped cheek muscles. The receiver unit, as shown in Fig 2c, connects to a PC and is responsible for retrieving data from the transmitter unit, wirelessly. These data are then mapped to appropriate system calls with the help of a device driver with customizable features, as shown in Fig 2d, enabling mouse control.

The *yaw* and the *pitch* angles are measured relative to a reference *zero* point, which is considered as the orientation of the user’s head at the device startup while the user focuses on the screen at its center. Therefore, at startup, the transmitter unit goes through a calibration phase (8−10 *seconds*), during which the user orients his/her head towards the middle of the screen and holds the position until they hear 3 consecutive beeps. The third beep at the end of the calibration phase indicates that the device has been calibrated, and the user is free to move his/her head or twitch cheek muscles for controlling the mouse. Furthermore, to enhance the user’s experience while interacting with the device, two gesture controls, one for *enabling* and the other for *disabling* the mouse functionality of the AMC, have been incorporated. For disabling the mouse, a user has to rotate his/her head down to about 35° and twitch both cheek muscles, after which they can easily interact with their surroundings. During this time, no data will be transmitted to the receiver unit. For enabling the mouse, the user has to rotate his head up to about 35° and twitch both cheek muscles, after which the mouse can be controlled as before.

## Hypothesis development and research model

In this section, in addition to the original psychological constructs of the TAM (PU, PEU, ATU, SN, and BI) [42], we discuss the three external constructs, namely—Personal Innovativeness (PI), Technology Anxiety (TA), and Perceived Behavioral Control (PBC), that may influence the acceptance of the proposed AMC technology by people with upper limb disability. Furthermore, we also state our hypotheses regarding how each of the constructs may influence other constructs, thereby, proposing our conceptual framework for validation.

### Constructs of the TAM

The primary objective of the TAM is to identify the psychological constructs that determine users’ attitude towards (*positive* or *negative*) and intention to accept a particular technology, identifying the direct or indirect influence of the constructs on one another in the process [37–39, 41–44, 47–57, 67]. TAM theorizes a direct positive effect of PU on the constructs ATU and BI [42]. In the context of our study, PU is the perception of a user with an upper limb disability that they will be able to interact with a computer while enhancing their productivity, work efficiency, etc. with the proposed AMC. Prior studies have analyzed similar influences in the adoption of wearable technologies as well [38, 39, 44, 45, 49–52, 67]. On the other hand, PEU of the AMC refers to the degree to which a user perceives that it allows effortless interaction mechanisms, consequently affecting their PU and ATU towards it [8, 38, 40–42, 52]. Furthermore, an easy-to-use technology supposedly should have a greater influence on the confidence (PBC) of using that technology [60]. As evident from the literature [37–39, 42–44, 47–56], SN is a crucial construct for determining user acceptance of any wearable technology. However, to the best of our knowledge, there are insignificant references to the analysis of the effect of SN and ATU on a user’s intention (BI) of using a wearable AMC in the literature [41, 50, 56, 86]. Therefore, we propose the following hypotheses -

**H1:** *PU has a positive influence on ATU of the wearable AMC*.
**H2:** *PU has a positive influence on BI of the wearable AMC*.
**H3:** *PEU has a positive influence on PU of the wearable AMC*.
**H4:** *PEU has a positive influence on PBC while using the wearable AMC*.
**H5:** *PEU has a positive influence on ATU of the wearable AMC*.
**H6:** *SN has a positive influence on PU of the wearable AMC*.
**H7:** *SN has a positive influence on PEU of the wearable AMC*.
**H8:** *SN has a positive influence on ATU of the wearable AMC*.
**H9:** *SN has a positive influence on a user’s BI of using the wearable AMC*.
**H10:** *ATU has a positive influence on a user’s BI of using the wearable AMC*.

### Technology Anxiety (TA)

Technology Anxiety (TA) as proposed by Lin et al. [38] and Tsai et al. [39], is the perceived fear involved with any technology. For reasons mentioned earlier, the potential anxiety factors about the proposed AMC could be the device ergonomics, complexity of interaction techniques, hygiene issues, etc. Logically, TA should be negatively correlated with PEU [38, 39]. Therefore, the following hypothesis is stated -

**H11:** *TA has a negative influence on PEU of the wearable AMC*.

### Perceived Behavioral Control (PBC)

As reported by previous studies [40–42, 54, 55, 60], the confidence while performing any task, or in other words, Perceived Behavioral Control (PBC), may have a direct or indirect positive impact on the ATU and BI of accepting a technology depending on the context. However, to the best of our knowledge, there are insignificant references to PBC in the context of users’ acceptability of the proposed wearable AMC. Therefore, we state the following hypotheses -

**H12:** *PBC has a positive influence on ATU of the wearable AMC*.
**H13:** *PBC has a positive influence on a user’s BI of using the wearable AMC*.

### Personal Innovativeness (PI)

Personal Innovativeness (PI) may be defined as, “*the presence of characteristics, such as—willingness, curiosity, search for novelty, creativity, etc. in an individual for adopting a technology*” [43–45]. Highly innovative individuals tend to be confident, and enthusiastic, and therefore, require a shorter time to accept a particular technology [43, 61]. Such individuals can realize the potential advantages of adopting a technology, earlier than others, and therefore, their positive ATU and BI towards that technology increase gradually [44, 45, 56, 61]. However, the literature suggests that the effect of PI on different constructs of the TAM in different contexts requires further investigation [62, 63]. In the context of this study, the proposed wireless head-mounted AMC may be considered as a technological innovation for people with upper limb disability in Bangladesh. Therefore, we postulated the following hypotheses -

**H14:** *PI has a positive influence on PU of the wearable AMC*.
**H15:** *PI has a positive influence on PEU of the wearable AMC*.
**H16:** *PI has a positive influence on PBC while using the wearable AMC*.
**H17:** *PI has a positive influence on ATU of the wearable AMC*.
**H18:** *PI has a positive influence on a user’s BI of using the wearable AMC*.

### Research model

**Fig 3:**
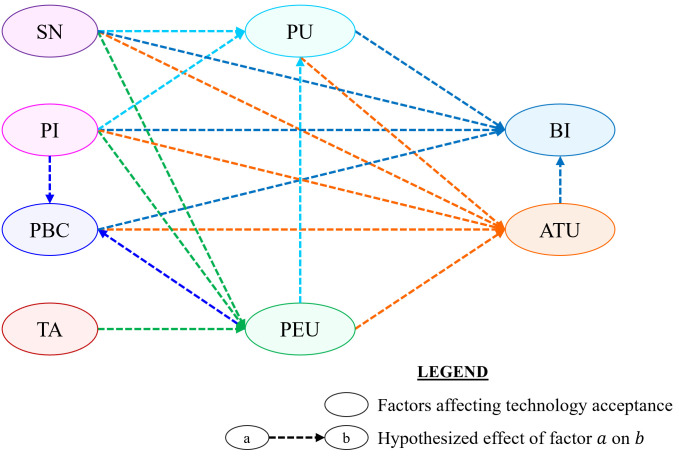
Modified Technology Acceptance Model (TAM) of the proposed Assistive Mouse Controller (AMC). [PU: Perceived Usefulness, PEU: Perceived Ease of Use, SN: Subjective Norm, PI: Personal Innovativeness, TA: Technology Anxiety, PBC: Perceived Behavioral Control, ATU: Attitude Towards Usage, and BI: Behavioral Intention].

In this study, 18 hypotheses (**H1-H18**) have been postulated for verifying the relationship among *eight* psychological constructs of the Technology Acceptance Model (TAM), namely—Perceived Usefulness (PU), Perceived Ease of Use (PEU), Subjective Norm (SN), Personal Innovativeness (PI), Technology Anxiety (TA), Perceived Behavioral Control (PBC), Attitude Towards Usage (ATU), and Behavioral Intention (BI), concerning the acceptance of the proposed AMC. Out of these 18 hypotheses, 7 of them were *conventional*, i.e. considered in the original framework of the TAM as well [40–42]. A summary of the postulated hypotheses and a TAM-based research model, specific to the adoption of the proposed AMC, are given in Table 1 and Fig 3, respectively. The validity of these hypotheses will be subsequently analyzed to identify the constructs that are significant in determining users’ acceptability of the proposed AMC from the socioeconomic perspective of Bangladesh.

**Table 1:** Summary of the postulated hypotheses for analyzing acceptability of the proposed AMC using the Technology Acceptance Model (TAM).

Influencing Construct	Hypothesis	Hypothesized Influence	Conventional?^*^
**Perceived Usefulness (PU)**	H1	PU→ATU^[+]^	**Yes**
	H2	PU→BI^[+]^	**Yes**
**Perceived Ease of Use (PEU)**	H3	PEU→PU^[+]^	**Yes**
	H4	PEU→PBC^[+]^	No
	H5	PEU→ATU^[+]^	**Yes**
**Subjective Norm (SN)**	H6	SN→PU^[+]^	**Yes**
	H7	SN→PEU^[+]^	No
	H8	SN→ATU^[+]^	No
	H9	SN→BI^[+]^	**Yes**
**Attitude Towards Usage (ATU)**	H10	ATU→BI^[+]^	**Yes**
**Technology Anxiety (TA)** ^a^	H11	TA→PEU^[−]^	No
**Perceived Behavioral Control (PBC)** ^a^	H12	PBC→ATU^[+]^	No
	H13	PBC→BI^[+]^	No
**Personal Innovativeness (PI)** ^a^	H14	PI→PU^[+]^	No
	H15	PI→PEU^[+]^	No
	H16	PI→PBC^[+]^	No
	H17	PI→ATU^[+]^	No
	H18	PI→BI^[+]^	No
Total conventional hypotheses			7
Total hypotheses (including conventional ones)			18

^[+]^Hypothesized *positive* influence.

^[−]^Hypothesized *negative* influence.

^a^Psychological constructs external to the original TAM framework considered in this study.

^*^A hypothesis is considered conventional, if it is also present in the original TAM[40–42].

## Research methodology

### Participants

The target respondents of this survey were individuals who despite having basic computing knowledge fail to interact with a computer due to any form of upper limb disability and are willing to adapt to an alternative modality for human-computer interaction. As mentioned earlier, analysis of the acceptance of technology using the TAM framework involves a self-administered, closed-ended survey questionnaire, which is divided into sections that represent different psychological constructs. However, due to the onset of COVID-19 at the time of this study, it was not possible for us to facilitate first-hand device usage through physical sessions with such individuals, and therefore, the survey had to be conducted online. Consequently, a comprehensive video of device interaction involving real-life users with upper limb disability (recorded prior to COVID-19 with verbal consent from them after the device prototype had been developed) was accommodated. This video helped demonstrate the prospect, usage, and interaction mechanism of the proposed AMC for executing different computing tasks to the respondents of the survey. Although familiarizing the users with the proposed AMC in this manner represents a notable limitation of our study, evidence of such an approach to TAM analysis can be found in the literature as well [87, 88].

The survey questionnaire was circulated only among those respondents, who verbally consented to participate in the survey, via email or social networking sites. In connection to this, a total of 150 individuals with stroke-induced upper limb disability, among which 107 were Male (71.33%, Mean Age: 33.13 ± 5.38 years) and 43 were Female (28.67%, Mean Age: 34.49 ± 4.12 years), participated in the online survey with the help of their caregivers. The survey was conducted between 01 January and 31 May 2022, and the corresponding data were accessed for research purposes between 01 June and 31 August 2022. The authors did not have access to information that could identify participants during or after the survey was conducted. The study was approved by the Department of Research, Extension, Advisory, Services, and Publications (REASP) at the Islamic University of Technology (IUT).

### Developing measurement items

**Fig 4:**
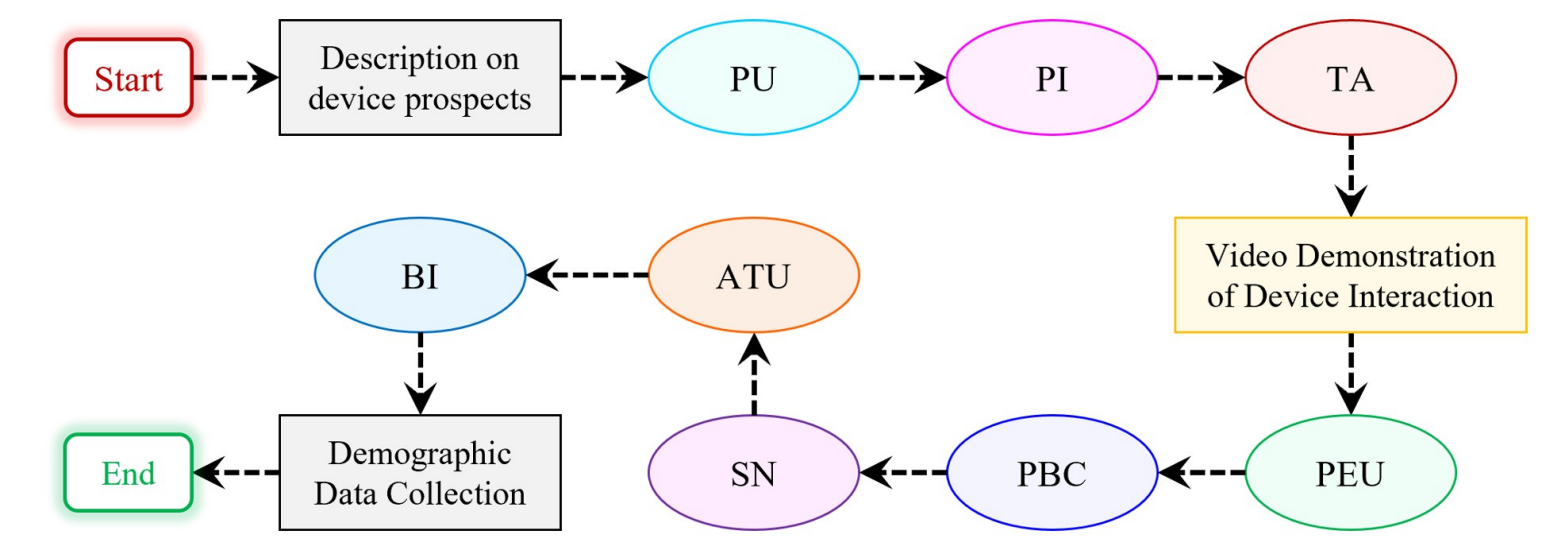
Organization of the constructs of the Technology Acceptance Model (TAM) in the online survey of the proposed Assistive Mouse Controller (AMC). [PU: Perceived Usefulness, PEU: Perceived Ease of Use, SN: Subjective Norm, PI: Personal Innovativeness, TA: Technology Anxiety, PBC: Perceived Behavioral Control, ATU: Attitude Towards Usage, and BI: Behavioral Intention].

In this study, 28 measurement items were considered for developing the construct measures, where few items were *adopted* and some were *adapted* from prior studies to suit the context of this study. Some items were *newly developed* specifically for this study as well. The questionnaire was subdivided into 11 sections, where 8 sections addressed items corresponding to the different psychological constructs (PU, PEU, SN, PI, TA, PBC, ATU, and BI) of the proposed research model, as shown in Fig 3. In 2 of the remaining 3 sections, a brief description of the prospects of the proposed AMC and questions relevant to demographic data (*name, age*, and *gender*) were presented. The remaining 1 section was allotted for the video demonstration of how different pointing [72] and typing tasks can be accomplished with the proposed AMC. The organization of the 11 sections of the questionnaire is depicted in Fig 4.

It is to be noted from this organization that the items corresponding to the constructs PU, PI, and TA are presented after the device description and before the video demonstration of device interaction. However, the items corresponding to the constructs PEU, PBC, SN, ATU, and BI, are presented after the video demonstration. Such organization of the questionnaire was made with the following objectives in mind -

(**a**) To record the respondents’ PI that might affect their ATU later on and to capture their initial thoughts on the constructs, PU and TA, from the device description.
(**b**) Since first-hand interaction with the proposed AMC could not be accommodated for the participants of this study, it was necessary that the respondents had a clear understanding of the working mechanism of the AMC for analyzing their acceptance of the technology using the TAM. In connection to this, the video demonstration of device interaction will assist them with the constructs, PEU, PBC, and SN, which in the end, would be reflected on their ATU and BI of accepting the proposed AMC that facilitates human-computer interaction for the people with upper limb disability.

As pointed out by Jonald L. Pimentel [64], Likert scales essentially quantify bipolar opinions (*positive* or *negative*) about a particular statement. However, the responses may be biased due to several reasons, e.g., respondents’ tendency to avoid extreme opinions (*central tendency bias*) [64]. To remove this type of bias, Likert scale items with an even number of options (4-point or 6-point), or in other words, items with no choice of *neutrality* are generally suggested for greater reliability of the responses [64, 89–91]. In connection to this, a 4-point Likert scale was used to quantify the responses to the psychological constructs of the proposed research model, where the Likert scale representations were as follows—1—*Strongly Disagree*, 2—*Disagree*, 3—*Agree*, and 4—*Strongly Agree*. The items, corresponding to different psychological constructs considered for this study, are summarized in Table 2.

**Table 2:** Measures of the constructs of Technology Acceptance Model (TAM).

Psychological Construct	Items	Descriptions
**Perceived Usefulness (PU)** [39, 41, 42, 52, 54–56, 66–68]	PU1	The ability to interact with a computer will improve work efficiency.^b^
	PU2	The ability to interact with a computer will improve productivity.^b^
	PU3	The ability to interact with a computer will make life more convenient.^b^
**Perceived Ease of Use (PEU)** [39, 41, 42, 52, 54–56, 67, 68]	PEU1	The device is easy to put on and off.^c^
	PEU2	The interaction mechanism of the device is adequate and easy.^b^
	PEU3	The device requires very less physical and mental effort to use.^b^
	PEU4	Overall, the device is easy-to-use.^a^
**Subjective Norm (SN)** [42, 50, 54–56, 61]	SN1	People who are important to me think that I should use the device.^a^
	SN2	People who influence my behavior think that I should use the device.^a^
**Attitude Towards Usage (ATU)** [39, 52, 54, 55, 66, 67]	ATU1	I think positively about the device when it comes to the possibility of improving Health-Related Quality of Life (HRQoL).^c^
	ATU2	I think positively about the device when it comes to the possibility of facilitating employment opportunities.^c^
	ATU3	I think positively about the device when it comes to the possibility of facilitating economic independence.^c^
	ATU4	I think positively about the device when it comes to the possibility of facilitating innovation process.^c^
	ATU5	I think that the ability to interact with a computer, like a healthy person, will have a positive effect on mental wellbeing.^c^
	ATU6	Overall, I have a positive attitude towards the usage of this device.^a^
**Technology Anxiety (TA)**^d^ [39]	TA1	I initially thought that the device would be uncomfortable as a wearable technology.^c^
	TA2	I initially thought that the device would be difficult to wear.^c^
	TA3	I initially thought that the device would not be adjustable to fit my head size.^c^
	TA4	I initially thought that the device would pose ergonomic issues.^c^
	TA5	I initially thought that the device would be costly.^b^
**Perceived Behavioral Control (PBC)**^d^ [54, 55]	PBC1	I am confident that I can easily interact with a computer using this device.^a^
	PBC2	I am confident that I can control my interaction with a computer using this device.^a^
**Personal Innovativeness (PI)**^d^ [44, 51, 55, 56, 61]	PI1	If I heard about a new interaction device, I would look for ways to experiment with it.^b^
	PI2	I like to experiment with the interaction devices that make my life easier.^b^
	PI3	I like to experiment with the devices that make my computer interaction interesting.^b^
**Behavioral Intention (BI)** [39, 44, 45, 52, 54, 55, 61, 66, 67]	BI1	I intend to use this device in the future.^a^
	BI2	I intend to use this device for performing basic computational tasks.^c^
	BI3	I intend to use this device for being self-reliant.^c^

^a^Items that were ***adopted*** from prior studies.

^b^Items that were ***adapted*** from prior studies.

^c^Items that were ***newly developed***, specifically for this study.

^d^Psychological constructs external to the original TAM framework considered in this study.

Before being used for online data collection, the questionnaire was reviewed by a group of 10 reviewers who had prior experience with wearable technology and did not have any upper limb disabilities. Before scrutinizing the comprehensibility and obscurity of the measuring items, they were initially told online about the goal of the study and the survey. They endorsed the questionnaire’s sectional organization and pointed out any slight formatting flaws; these were subsequently corrected, and the questionnaire was approved for data collection.

### Data analysis

Before conducting CFA for assessing the reliability and validity of the corresponding measurement model, an adequacy test was performed to verify whether the sample size (*n* = 150) is suitable for CFA. Structural Equation Modeling (SEM) was then performed for hypotheses (**H1-H18**) testing and validation in *python* language with the help of the library, “*semopy*” [92], following the GLS method [77]. Consequently, a path model was generated summarizing the results of SEM [39, 50, 52, 56, 61, 66–70]. The *R*^2^-values of regression analysis were used to quantify the percentage of variance explained by the predictor variables in the proposed research model. Finally, the relative fit of the data to the model was analyzed using different fit indices (*χ*^2^/df, GFI, AGFI, CFI, TLI, NFI, RMSEA) [49, 67, 69, 80, 81].

## Results

### Measurement model

The adequacy test of the sample, considered for this study, was conducted using both Bartlett’s test of sphericity and Kaiser-Meyer-Olkin (KMO) measure of sampling adequacy [69, 70]. The sphericity test (*χ*^2^ = 2016.17, *p* < 0.001) indicated that the inter-construct correlation matrix was not an identity matrix, as shown in Table 5. The KMO value for the sample (*KMO* = 0.8057) indicated that the sample size was “*Adequate*” for Confirmatory Factor Analysis (CFA). A summary of the sample adequacy test results is given in Table 3.

**Table 3:** Summary of the sample adequacy testing for Confirmatory Factor Analysis (CFA).

Adequacy Test	Recommended Value	Sample Adequacy Test Value	Remark
Bartlett’s test of sphericity [69, 70]	Large *χ*^2^ value at *p*<0.05	*χ*^2^ = 2016.17, *p*<0.001	Inter-construct correlation matrix is not an identity matrix.
Kaiser-Meyer-Olkin (KMO) measure of sample size adequacy [69, 70]	Adequate (0.80 ≤ KMO < 1.00) Middling (0.70 ≤ KMO < 0.79) Mediocre (0.60 ≤ KMO < 0.69) Inadequate (KMO < 0.60)	0.8057	Adequate

The results of the measurement model, obtained using CFA, as shown in Table 4, indicated that the measurement items demonstrated strong psychometric properties. The internal reliability of the items in each of the psychological constructs, measured using Cronbach’s Alpha (CA) [39, 66], ranged between 0.7248 and 0.8969 and the overall reliability of the questionnaire was found to be 0.8801, which indicates “*Good*” reliability. In simple terms, the items could quantify the constructs accurately. The factor loadings (λ), as a measure of individual item reliability, were obtained using Principal Component Analysis with “*varimax*” rotation [68, 70]. The values of λ for 50% of the items ranged between 0.5 and 0.7, while the rest were above 0.7, both of which are permissible [39, 49, 61, 68, 69, 71]. In connection with these results, the reliability of the constructs can be ascertained.

**Table 4:** Reliability and Convergent Validity (CV) of the measurement model of users’ acceptance of the proposed Assistive Mouse Controller (AMC) (*n* = 150).

Psychological Construct	Items	Reliability		Individual Item Reliability	Internal Consistency and Convergent Validity (CV)		
		*Cronbach*’s Alpha (CA)	*Remark*	*Factor Loading (λ)*	*Composite Reliability (CR)*	*Average Variance Extracted (AVE)*	*Remark*
Perceived Usefulness (PU)	PU1	0.7248	Acceptable	0.7924	0.7293	0.4789	Acceptable
	PU2			0.7170			
	PU3			0.5428			
Perceived Ease of Use (PEU)	PEU1	0.7903	Acceptable	0.7876	0.7896	0.4858	Acceptable
	PEU2			0.6269			
	PEU3			0.6654			
	PEU4			0.6980			
Subjective Norm (SN)	SN1	0.8969	Good	0.8284	0.8016	0.6690	Good
	SN2			0.8073			
Attitude Towards Usage (ATU)	ATU1	0.8375	Good	0.5126	0.8224	0.4384	Acceptable
	ATU2			0.7233			
	ATU3			0.7110			
	ATU4			0.6519			
	ATU5			0.6883			
	ATU6			0.6636			
Technology Anxiety (TA)^a^	TA1	0.7423	Acceptable	0.6373	0.8192	0.4759	Acceptable
	TA2			0.6792			
	TA3			0.7141			
	TA4			0.7202			
	TA5			0.8073			
Perceived Behavioral Control (PBC)^a^	PBC1	0.7379	Acceptable	0.7532	0.6821	0.5181	Acceptable
	PBC2			0.6848			
Personal Innovativeness (PI)^a^	PI1	0.7602	Acceptable	0.7859	0.8109	0.5890	Good
	PI2			0.7992			
	PI3			0.7145			
Behavioral Intention (BI)	BI1	0.8396	Good	0.6776	0.7575	0.5110	Good
	BI2			0.6861			
	BI3			0.7767			
**Overall Reliability**		0.8801	Good	**Overall Convergent Validity**			Satisfactory

^a^Psychological constructs external to the original TAM framework considered in this study.

The internal consistency of the psychological constructs was measured using Composite Reliability (CR) [39, 61, 66, 68, 71] with values ranging between 0.7293 and 0.8224, which is indicative of “*Good*” internal consistency. However, for the construct Perceived Behavioral Control (PBC), the value of CR was found to be 0.6821, which is also acceptable [66, 71]. The validity of the measurement model was tested using CV [39, 70, 71, 73] and DV [39, 68]. For evaluating the CV, both CR and AVE were considered [39, 70, 71, 73]. *Five* out of the *eight* constructs (PU, TA, PBC, PEU, and ATU), considered in this study exhibited “*Acceptable*” CV with AVE ranging between 0.4384 and 0.5181, and CR ranging between 0.6821 and 0.8224. The remaining three constructs (SN, PI, and BI) had AVE ranging between 0.5110 and 0.6690, and CR ranging between 0.7575 and 0.8109, suggesting “*Good*” CV. Overall, the model had satisfactory CV. On the other hand, the DV of the constructs was evaluated using the Fornell and Larcker criterion [45]. The DV of the constructs of this study along with the Mean and the Standard Deviation (SD) are summarized in Table 5. It can be seen from this table that for each of the constructs, the corresponding squared root of AVE is greater than all the corresponding inter-construct correlations. Therefore, it can be stated that the model demonstrates satisfactory DV.

**Table 5:** The Mean, Standard Deviation (SD) and Discriminant Validity (DV) of the measurement model on users’ acceptance of the proposed Assistive Mouse Controller (AMC) (*n* = 150).

	Mean	SD	PU	TA^b^	SN	PBC^b^	PEU	PI^b^	ATU	BI	Remark on DV
**PU**	3.63	0.55	**0.6920** ^a^	-	-	-	-	-	-	-	Good
**TA^b^**	2.85	0.88	0.1680	**0.6899** ^a^	-	-	-	-	-	-	Good
**SN**	3.22	0.87	0.3990	0.1550	**0.8179** ^a^	-	-	-	-	-	Good
**PBC^b^**	3.57	0.59	0.3515	0.0246	0.2396	**0.7198** ^a^	-	-	-	-	Good
**PEU**	3.27	0.70	0.4282	-0.0700	0.3005	0.4965	**0.6970** ^a^	-	-	-	Good
**PI^b^**	3.63	0.60	0.3455	0.0649	0.2655	0.2886	0.1045	**0.7675** ^a^	-	-	Good
**ATU**	3.60	0.56	0.5614	0.1398	0.4418	0.4981	0.4944	0.3504	**0.6621** ^a^	-	Good
**BI**	3.50	0.72	0.4560	0.1652	0.6435	0.4233	0.3255	0.3013	0.5581	**0.7148** ^a^	Good

^a^ Squared root of the Average Variance Extracted (AVE); values below the diagonal are inter-construct correlations.

^b^ Psychological constructs external to the original TAM framework considered in this study.

### Structural model

**Fig 5:**
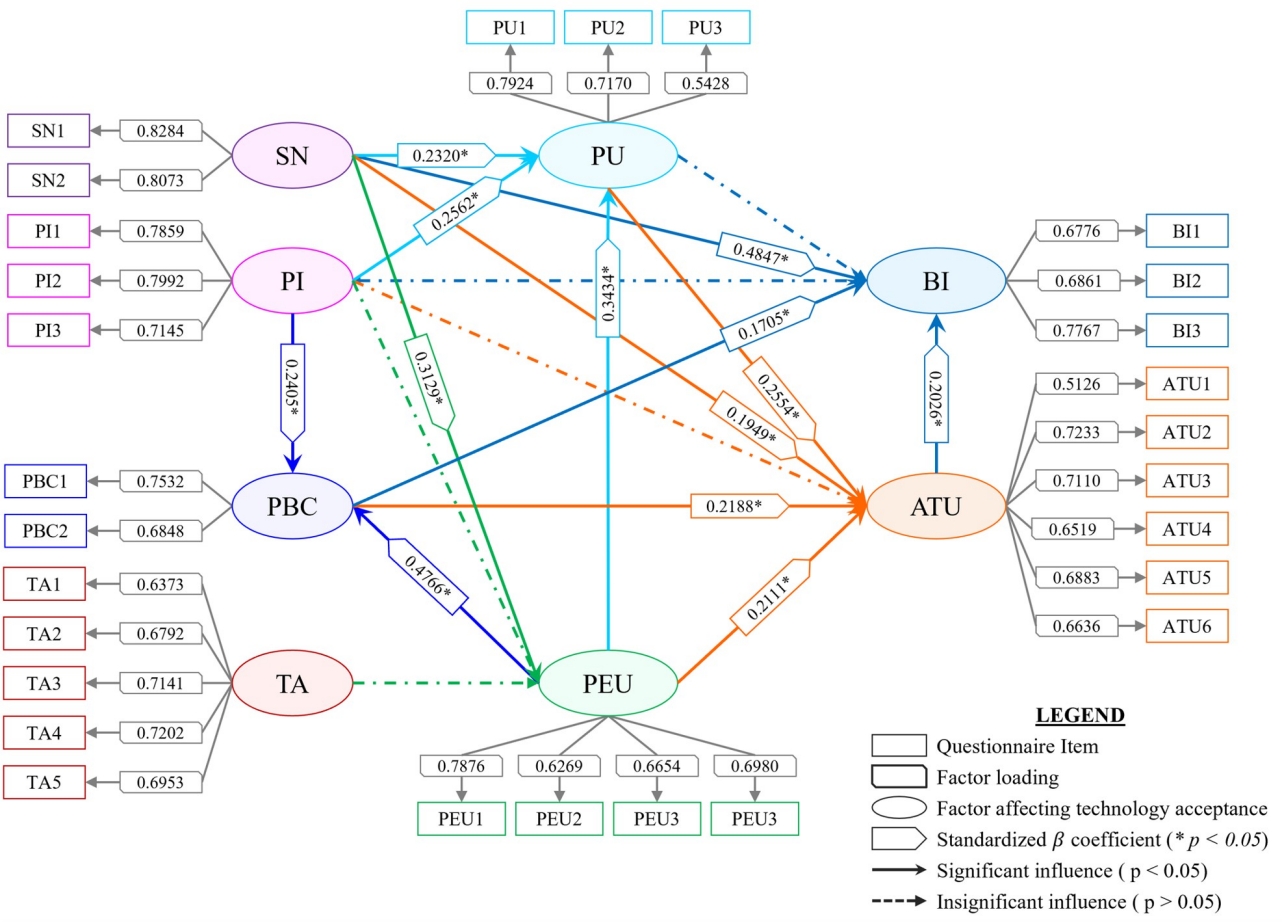
Final Technology Acceptance Model (TAM) of the proposed Assistive Mouse Controller (AMC). Structural Equation Modelling (SEM) was used for this purpose. The model shows standardized *β*-coefficients of the significant influences only.

**Fig 6:**
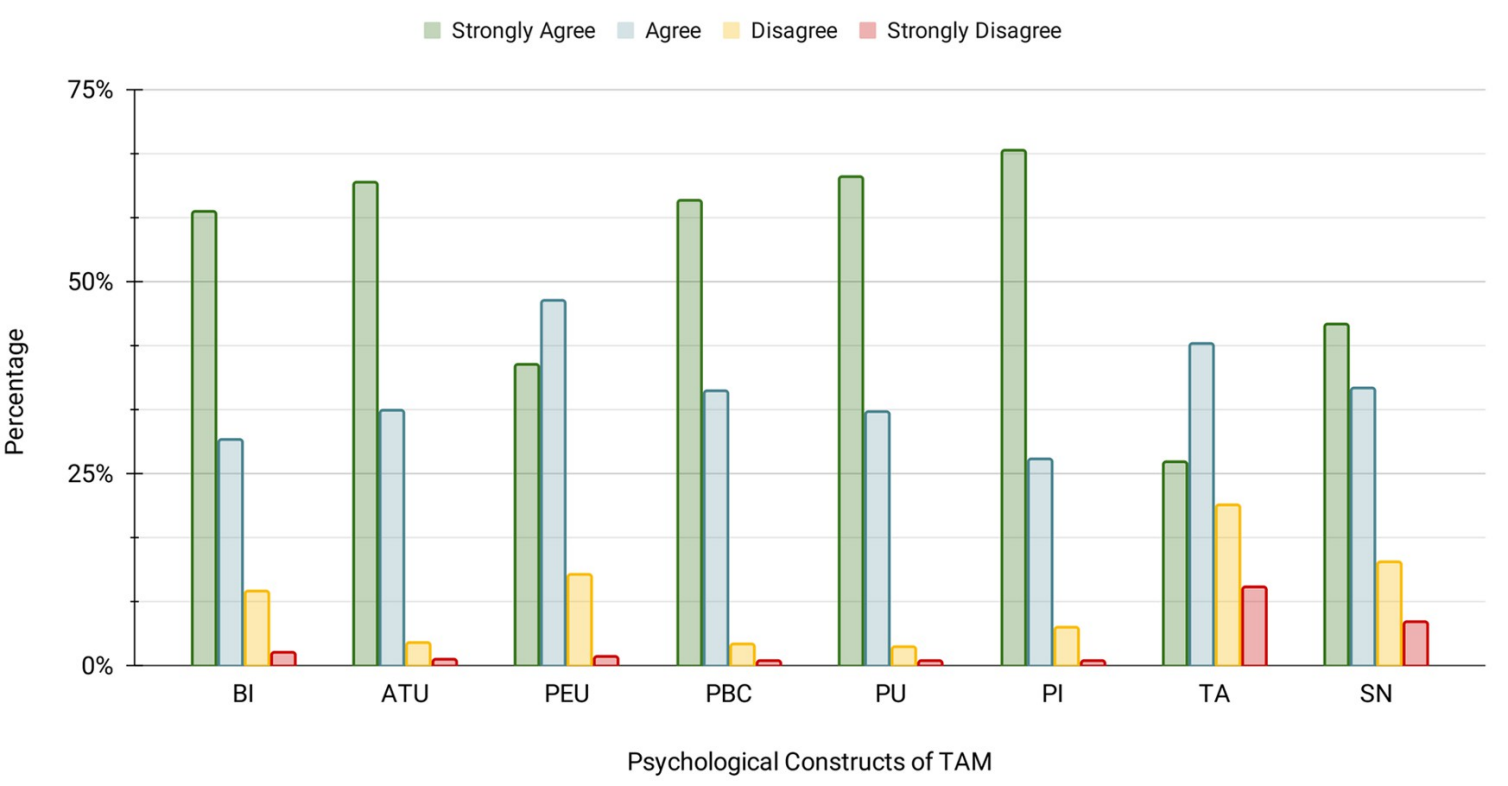
Average distribution of participants’ responses to the TAM questionnaire. [PU: Perceived Usefulness, PEU: Perceived Ease of Use, SN: Subjective Norm, PI: Personal Innovativeness, TA: Technology Anxiety, PBC: Perceived Behavioral Control, ATU: Attitude Towards Usage, and BI: Behavioral Intention].

From the results of SEM, as shown in Table 6, it was observed that 6 (85.71%) out of the 7 conventional hypotheses and 13 (72.22%) out of the total 18 hypotheses were supported. According to the order in which the measurement items were presented in the online survey questionnaire, as shown in Fig 4, about 94.16% of the participants (n = 150) claimed to have an innovative mindset (PI), and about 94.71% of them could perceive the usefulness (PU) of the proposed AMC from the description of the corresponding device alone. Analysis showed that before watching the video demonstration of device interaction, 68.61% of the participants reported feeling nervous (TA) about the AMC. However, after watching the video, about 86.86% of them were able to better perceive its ease of use (PEU) and 96.35% of them reported feeling confident (PBC) about using it to complete a computing activity. This can be validated from the structural model in Fig 5, as both PEU and PI had significant positive influences on PU and PBC (the hypothesis **H3**, **H4**, **H14**, and **H16** were supported). Furthermore, the constructs PU, PEU, and PBC had significant positive influences on ATU, supporting the hypotheses **H1**, **H5**, and **H12**, respectively. The participants claimed that they were encouraged to use the AMC by their peers (SN) in about 80.65% of the situations. Because of these reasons and how they perceived the AMC, most of them (about 96.44%) had a favorable attitude (ATU) toward it. Additionally, almost 88.56% of them indicated a positive intention (BI) to incorporate it into their way of life. Although prior studies have reported PU to have a direct significant effect on BI, the same was not observed in the context of this study, and therefore **H2** was not supported, which aligns with evidence found in the literature [39]. However, the constructs ATU and PBC were found to have significant positive influences on BI, supporting the hypotheses **H10** and **H13**, respectively. The motivation from their peers may have contributed to them being able to perceive the usefulness and ease of using the AMC better, while enhancing their positive attitude towards and intention to accept it. Consequently, SN was found to have significant positive influences on the constructs PU, PEU, ATU, and BI (the hypotheses **H6**, **H7**, **H8**, and **H9** were supported, respectively). These findings are consistent with prior studies related to technology adoption [39, 44, 49, 54, 60, 66]. Both of the constructs TA and PI had insignificant influences on PEU (the hypotheses **H11** and **H15** were not supported, respectively) while maintaining consistency with the results of prior studies [38, 39] as well. Furthermore, PI did not have any significant positive influence on ATU and BI of the AMC (the hypotheses **H17** and **H18** were not supported, respectively) as well. Fig 6 depicts the average distribution of participant responses for each of the psychological constructs considered in this study. The TAM path model of the proposed research model using SEM analysis is given in Fig 5.

**Table 6:** Summary of hypothesis test results using Structural Equation Modeling (SEM) (*n* = 150).

Influencing Construct	Hypothesis	Hypothesized Influence^a^	*β* coefficient	Standard Error	*z*-value^a^	*p*-value^a^	Support^a^
**PU**	H1	PU→ATU^[+]^*	0.2554	0.0725	3.5382	0.004	Yes
	H2	PU→BI^[+]^*	-0.0658	0.0697	0.9497	0.3423	**No**
**PEU**	H3	PEU→PU^[+]^*	0.3434	0.0684	4.9161	<0.0001	Yes
	H4	PEU→PBC^[+]^	0.4766	0.0679	6.9835	<0.0001	Yes
	H5	PEU→ATU^[+]^*	0.2111	0.0733	2.8306	0.0046	Yes
**SN**	H6	SN→PU^[+]^*	0.2320	0.0704	3.2200	0.0013	Yes
	H7	SN→PEU^[+]^	0.3129	0.0807	3.8678	0.0001	Yes
	H8	SN→ATU^[+]^	0.1949	0.0646	2.9600	0.0031	Yes
	H9	SN→BI^[+]^*	0.4847	0.0632	7.5402	<0.0001	Yes
**ATU**	H10	ATU→BI^[+]^*	0.2026	0.0759	2.6737	0.0075	Yes
**TA^b^**	H11	TA→PEU^[−]^	-0.1217	0.0780	-1.5577	0.1193	**No**
**PBC^b^**	H12	PBC→ATU^[+]^	0.2188	0.0699	3.0913	0.0020	Yes
	H13	PBC→BI^[+]^	0.1705	0.0638	2.6417	0.0083	Yes
**PI^b^**	H14	PI→PU^[+]^	0.2562	0.0675	3.7101	0.0002	Yes
	H15	PI→PEU^[+]^	0.0295	0.0799	0.3683	0.7126	**No**
	H16	PI→PBC^[+]^	0.2405	0.0678	3.5241	0.0004	Yes
	H17	PI→ATU^[+]^	0.1293	0.0648	1.9578	0.0502	**No**
	H18	PI→BI^[+]^	0.0326	0.0611	0.5249	0.5997	**No**
Total conventional hypotheses							7
Number of conventional hypotheses supported							6 (85.71%)
Number of conventional hypotheses not supported							1 (14.29%)
Total hypotheses (including conventional ones)							18
Number of hypotheses supported (including conventional ones)							13 (72.22%)
Number of hypotheses not supported (including conventional ones)							5 (27.78%)

^[+]^Hypothesized *positive* influence.

^[−]^Hypothesized *negative* influence.

^a^A path was considered significant, if either *z*< -1.96 or *z*> 1.96 and *p*<0.05.

^b^ Psychological constructs external to the original TAM framework considered in this study.

^*^A conventional hypothesis, which is also present in the original Technology Acceptance Model (TAM) [40–42].

After the hypotheses were tested, the explanatory power of the research model was assessed using the *R*^2^-values of regression analysis. Prior studies have stated that *R*^2^-values greater than 0.67, 0.33, and 0.19 can be termed, “*substantial*”, “*moderate*”, and “*weak*”, respectively [66]. It was found that the proposed model explained about 49.02% of the variation in users’ Attitude Towards Usage (ATU) and 53.71% of the variation in their Behavioral Intention (BI) to accept the proposed AMC for their interaction with a computer. The corresponding *R*^2^-values, along with the *F*-statistics and the corresponding *p*-value of the other constructs, ATU, PU, PEU, and PBC are reported in Table 7. It can be observed from this table that all the predictions were significant at *p* ≤ 0.001. Furthermore, the constructs TA, SN, and PI altogether, explained about 10.50% of the variation in PEU, with SN being a significant predictor. Although the *R*^2^-value of PEU was very low compared to the other constructs, it was statistically significant at *p* = 0.001 and exceeded the recommended benchmark, which requires *R*^2^ being greater than 0.10 [45, 51]. It can also be observed from Table 7 that the *R*^2^ values exhibited an increasing trend as the model proceeds towards determining BI of accepting the proposed AMC, which indicated that the predictors of the research model were adequate in the context of this study. In connection to this, it can be established that the model explained an acceptable variation in the predicted constructs—PU, PEU, PBC, ATU, and BI. Furthermore, the relative fit of the structural model was analyzed using various fit indices (e.g., *χ*^2^/df, GFI, AGFI, CFI, TLI, NFI, RMSEA), as shown in Table 8. It is evident from this table that the values of all the indices were consistent with their recommended threshold values [49, 67, 69, 80, 81, 93–100]., which suggest a good model fit.

**Table 7:** Summary of *R*^2^ statistic of the psychological constructs.

Predicted Construct	Predictor Constructs	*R*^2^-Value	*F*-stat	*p*-value
**BI**	**ATU**^a^, PU, **PBC**^a^^b^, **SN**^a^, PI^b^	0.5371	33.4171	<0.0001
**ATU**	**PU**^a^, **PEU**^a^, **PBC**^a^^b^, **SN**^a^, PI^b^	0.4902	27.6876	<0.0001
**PU**	**PEU**^a^, **SN**^a^, **PI**^a^^b^	0.3212	23.0332	<0.0001
**PBC** ^b^	**PEU**^a^, **PI**^a^^b^	0.3031	31.9698	<0.0001
**PEU**	TA, **SN**^a^, PI^b^	0.1050	5.7121	0.0010

^a^ Significant predictors at *p*<0.05.

^b^ Psychological constructs external to the original TAM framework considered in this study.

**Table 8:** Structural model fit analysis (*n* = 150).

Fit Indices	Recommended Value	Research Model	Remark
***χ*^2^/df**	≤3.00	1.1987	Good Fit
**Goodness-of-Fit Index (GFI)**	≥0.90	0.9610	Good Fit
**Adjusted GFI (AGFI)**	≥0.80	0.9070	Good Fit
**Comparative Fit Index (CFI)**	≥0.90	0.9930	Good Fit
**Tucker-Lewis Index (TLI)**	≥0.90	0.9833	Good Fit
**Normed Fit Index (NFI)**	≥0.90	0.9610	Good Fit
**Root-Mean-Square Error of Approximation (RMSEA)**	≤0.08	0.0365	Good Fit

## Discussion

The purpose of this study was to develop a working prototype of a sensor-based head-mounted Assistive Mouse Controller (AMC), followed by a TAM-based analysis of its acceptability to the people with upper limb disabilities from the socioeconomic perspective of Bangladesh.

To reduce the risk of an ineffective and impractical prototype design, it is imperative to adopt a user-centered design approach [101] while considering their inputs to get a proper understanding of their requirements. However, due to the onset of COVID-19, it was challenging for us to recruit individuals or focus groups to carry out primary user-based requirement analysis through face-to-face interview sessions. Nonetheless, we were able to overcome this challenge by analyzing prior research [8] for specific design principles as part of user requirements in such contexts. Initially, we considered the eye wink gesture for actuating mouse clicks. However, long-term interaction with a computer involving frequent eye wink gestures can cause eye strain, leading to headaches [102]. Considering the long-term health issues of this gesture, it was challenging for us to consider an alternative generic facial gesture for this purpose. However, after rigorous analysis and brainstorming sessions, and with motivation from prior research [103], we adopted cheek muscle twitches for actuating mouse clicks using the proposed AMC.

For analyzing users’ acceptability of the proposed AMC, 18 hypotheses were postulated, which involved the 5 original constructs of TAM (PU, PEU, SN, ATU, and BI) along with 3 external constructs relevant to the socioeconomic perspective of Bangladesh (TA, PBC, and PI). To the best of our knowledge, the TAM-based acceptability analysis of an AMC is the first of its kind in such a context. The proposed TAM model can explain about, 49.02% and 53.71% of the variation in users’ positive attitude and intention to adopt the proposed AMC, respectively. Since the use of ATs is not widespread in Bangladesh let alone that of an AMC [13–16], it is natural for the people with upper limb disabilities in Bangladesh to exhibit signs of *anxiety*, *lack of confidence*, and *innovation* about the acceptance of the proposed AMC aimed towards equitable human-computer interaction. However, the users may be able to overcome these psychological shortcomings if first-hand interaction with it can be ensured through a trial usage period, subject to further investigation. Considering the beta coefficients of the positive significant influences of different psychological constructs on the participants’ attitude towards using the AMC (as seen from Table 6), they must be able to perceive its usefulness first, followed by its ease of use, and lastly, receive support, education, and motivation from the society to be able to interact with a computer for improving their socioeconomic status. Therefore, if proper initiatives to motivate, educate, and train them are taken by different government and/or private organizations in Bangladesh, the acceptability of the proposed AMC by the stakeholders may be ensured with a higher probability of success [37]. However, based on our findings, we may safely state that the participants endorsed the easy, simple, and intuitive interaction mechanisms of the AMC, which along with their innovative mindset, and support from their peers enabled them to perceive its usefulness better while enhancing their confidence in and attitude towards using it for carrying out a computing task.

Although our findings suggest that the current prototype of the AMC is potentially highly acceptable to the stakeholders, one major limitation of our study is that they could not interact with the AMC firsthand due to the onset of COVID-19, and therefore, they might not have perceived the practical implications of its usage just by watching a video demonstration of device interaction. This implies that the results of the TAM analysis are specific to this study and may not hold for a different wearable AMC technology in a different context. Therefore, future research may be focused on improving the explanatory power of the same or a different model with the same or an advanced prototype of the AMC, while facilitating first-hand device interaction, considering a larger sample size, or exploring a wide range of psychological constructs that were not considered in this study, such as—perceived enjoyment, perceived ubiquity, pricing, facilitating conditions, aesthetics, resistance to change, compatibility, etc. Furthermore, since actual usage of the proposed AMC could not be facilitated for the participants of the study, we were unable to measure the influence of users’ positive BI to use the proposed device on its actual usage. Therefore, we express our keen interest to explore this research avenue through a longitudinal cohort study in the future. Another potential future avenue of research may be to understand how a user adapts to performing specific tasks with the device over time, by measuring their decrease in reaction times. In this regard, the power law of practice [104] may be utilized, according to which the logarithm of response time for a certain task decreases linearly with the logarithm of practice trials encountered. Furthermore, we intend to analyze and assess the usability [101, 105–107] of the proposed AMC, while leveraging the System Usability Scale (SUS) in the future. It may help us modify existing gestures or add novel ones so that both the disabled community and the academic research body can benefit from its usage.

To summarize, the findings of this study suggest that for the proposed AMC, in addition to perceived usefulness, ease of use, and positive social influence, a high level of confidence owing to the easier working mechanism of the device and highly innovative personality significantly influences positive attitude and intention towards using the device from the socioeconomic perspective of Bangladesh.
